# 4-[(5R*,10bR*)-2-Methyl-1,10b-dihydro­pyrazolo[1,5-*c*][1,3]benzoxazin-5-yl]benzoic acid

**DOI:** 10.1107/S1600536808003607

**Published:** 2008-02-06

**Authors:** Viktor Kettmann, Jan Světlík

**Affiliations:** a Faculty of Pharmacy, Comenius University, Odbojarov 10, SK-83232 Bratislava, Slovakia

## Abstract

In the title compound, C_18_H_16_N_2_O_3_, a potential inhibitor of the cyclo­oxygenase-2 isoenzyme, the pyrazoline ring exists in a flattened envelope conformation with one C atom deviating by 0.463 Å from the mean plane of the remaining four atoms. The puckering of the central oxazine ring is more severe, with one N atom and one C atom displaced by 0.235 (6) and 0.370 (2) Å, respectively, on opposite sides of the mean plane defined by the other four atoms; the conformation is that of a half-chair. As a result, the mol­ecule as a whole is not planar. The carboxyl group is involved in an inter­molecular O—H⋯N hydrogen bond, which links the mol­ecules into centrosymmetric dimers.

## Related literature

For related literature, see: Palomer *et al.* (2002 ▶); Subbaramaiah *et al.* (2002 ▶); Světlík *et al.* (2005 ▶).
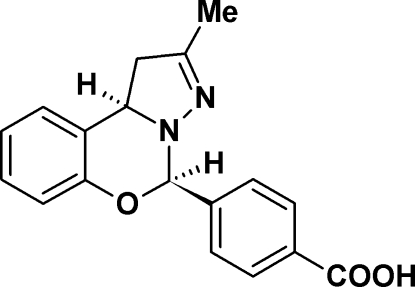


## Experimental

### 

#### Crystal data

C_18_H_16_N_2_O_3_
*M* *_r_* = 308.33
Triclinic, 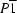

*a* = 6.638 (2) Å
*b* = 10.997 (3) Å
*c* = 11.141 (3) Å
α = 70.78 (2)°
β = 80.85 (3)°
γ = 79.15 (2)°
*V* = 750.1 (4) Å^3^
*Z* = 2
Mo *K*α radiation
μ = 0.09 mm^−1^
*T* = 296 (2) K
0.30 × 0.20 × 0.15 mm

#### Data collection

Siemens P4 diffractometer
Absorption correction: none
5341 measured reflections
4312 independent reflections
3216 reflections with *I* > 2σ(*I*)
*R*_int_ = 0.019
3 standard reflections every 97 reflections intensity decay: none

#### Refinement

*R*[*F* ^2^ > 2σ(*F* ^2^)] = 0.051
*wR*(*F* ^2^) = 0.152
*S* = 1.01
4312 reflections
210 parameters
H-atom parameters constrained
Δρ_max_ = 0.29 e Å^−3^
Δρ_min_ = −0.22 e Å^−3^

### 

Data collection: *XSCANS* (Siemens, 1991 ▶); cell refinement: *XSCANS*; data reduction: *XSCANS*; program(s) used to solve structure: *SHELXS97* (Sheldrick, 2008 ▶); program(s) used to refine structure: *SHELXL97* (Sheldrick, 2008 ▶); molecular graphics: *PLATON* (Spek, 2003 ▶); software used to prepare material for publication: *SHELXL97*.

## Supplementary Material

Crystal structure: contains datablocks global, I. DOI: 10.1107/S1600536808003607/bi2280sup1.cif

Structure factors: contains datablocks I. DOI: 10.1107/S1600536808003607/bi2280Isup2.hkl

Additional supplementary materials:  crystallographic information; 3D view; checkCIF report

## Figures and Tables

**Table 1:** Hydrogen-bond geometry (Å, °)

*D*—H⋯*A*	*D*—H	H⋯*A*	*D*⋯*A*	*D*—H⋯*A*
O8—H8*A*⋯N3^i^	0.82	1.93	2.7356 (17)	168

Symmetry code: (i) 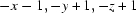.

**Fig. 1.**
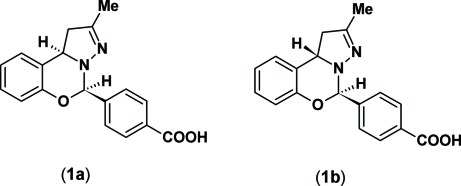
The cis title compound (1a) and its trans isomer (1 b).

**Fig. 2.**
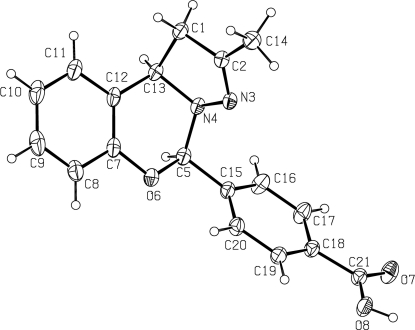
Molecular structure with displacement ellipsoids drawn at 35% probability for non-H atoms.

## supplementary crystallographic information

### Comment

Recently, based on a pharmacophoric model of the cyclooxygenase-2 (COX-2) inhibitors (Palomer *et al.*, 2002) as novel anticancer drugs (Subbaramaiah *et al.*, 2002), we designed and prepared a series of 2- and 5-substituted derivatives containing the tricyclic system shown in Fig. 1. In order to recognize enzyme binding requirements for this fused heterocycle, we selected the 2-methyl-5-carboxyphenyl derivative, for single-crystal X-ray analysis. The compound was obtained as a 1:1 mixture of the *cis* (1a) and *trans* (1 b) diastereomers and we report here the structure of the *cis* isomer.

The molecular structure and atom-numbering scheme is shown in Fig. 2. Bond distances and angles are close to those generally expected. The atom O6 is essentially *sp*^2^-hybridized and involved in conjugation with the benzo ring as indicated by the valence angle at this atom and a non-equivalency of the O6—C5 and O6—C7 bonds.

The most interesting feature of the structure—the spatial relationship between the pharmacophoric elements (hydrophobic groups and H-bond donors/acceptors)—is given by conformation of the (partially) saturated rings. Thus, the pyrazoline ring adopts a flat-envelope conformation with atom C13 (at the flap) deviating by 0.463 Å from the mean plane of the remaining atoms. The central oxazine ring is also non-planar and is puckered in such a manner that the four atoms O6, C7, C12 and C13 are planar to within 0.006 (2) Å, while atoms N4 and C5 are displaced by 0.235 (6) and 0.370 (2) Å, respectively, on opposite sides of the plane. As a result of the relatively severe puckering of the central ring, the molecule as a whole is non-planar but consists of two approximately planar segments, C5/O6/C7–C13 and C13/C1/C2/N3/N4/C5, folded about the C5···C13 line [dihedral angle 71.7 (1)°]. The carboxyphenyl substituent is rotated by 39.8 (1)° from the mean plane of the oxazine ring.

The crystal packing is dominated by a hydrogen bond between centrosymmetrically related molecules (Table 1) which result in formation of hydrogen-bonded dimers.

### Experimental

Synthesis of the title compound has been described previously (Světlík *et al.*, 2005). In short, a solution of 4-carboxybenzaldehyde (0.30 g, 2 mmol) and pyrazoline (0.35 g, 2 mmol) in ethyl acetate (14 ml) and methanol (1 ml) was left to react at room temperature for 1 h. The resulting precipitate was filtered off and crystallized from ethanol to obtain (1a) (70% yield; m.p. 474–480 K) as colourless crystals. Crystals suitable for the X-ray analysis were obtained by slow crystallization from acetone.

### Refinement

H atoms were visible in difference maps, but were placed geometrically and subsequently treated as riding atoms with distances C—H = 0.93 Å (CH_arom_), 0.97 (CH_2_) or 0.98 Å (CH) and 0.96 Å (CH_3_) and O—H = 0.82 Å (COOH); *U*_iso_ of the H atoms were set to 1.2 (1.5 for the methyl and carboxy H atoms) times *U*_eq_ of the parent atom.

### Crystal data

**Table:**

C_18_H_16_N_2_O_3_	*Z* = 2
*M**_r_* = 308.33	*F*_000_ = 324
Triclinic, *P*1	*D*_x_ = 1.365 Mg m^−^^3^
Hall symbol: -P 1	Melting point: 477 K
*a* = 6.638 (2) Å	Mo *K*α radiation λ = 0.71073 Å
*b* = 10.997 (3) Å	Cell parameters from 20 reflections
*c* = 11.141 (3) Å	θ = 7–18º
α = 70.78 (2)º	µ = 0.09 mm^−^^1^
β = 80.85 (3)º	*T* = 296 (2) K
γ = 79.15 (2)º	Prism, colourless
*V* = 750.1 (4) Å^3^	0.30 × 0.20 × 0.15 mm

### Data collection

**Table:**

Siemens P4 diffractometer	*R*_int_ = 0.019
Radiation source: fine-focus sealed tube	θ_max_ = 30.0º
Monochromator: graphite	θ_min_ = 2.0º
*T* = 296(2) K	*h* = −1→9
ω/2θ scans	*k* = −14→14
Absorption correction: none	*l* = −15→15
5341 measured reflections	3 standard reflections
4312 independent reflections	every 97 reflections
3216 reflections with *I* > 2σ(*I*)	intensity decay: none

### Refinement

**Table:**

Refinement on *F*^2^	Secondary atom site location: difference Fourier map
Least-squares matrix: full	Hydrogen site location: inferred from neighbouring sites
*R*[*F*^2^ > 2σ(*F*^2^)] = 0.051	H-atom parameters constrained
*wR*(*F*^2^) = 0.152	*w* = 1/[σ^2^(*F*_o_^2^) + (0.0853*P*)^2^ + 0.0987*P*] where *P* = (*F*_o_^2^ + 2*F*_c_^2^)/3
*S* = 1.01	(Δ/σ)_max_ = 0.001
4312 reflections	Δρ_max_ = 0.29 e Å^−^^3^
210 parameters	Δρ_min_ = −0.22 e Å^−^^3^
Primary atom site location: structure-invariant direct methods	Extinction correction: none

### Special details

**Table:**

Geometry. All e.s.d.'s (except the e.s.d. in the dihedral angle between two l.s. planes) are estimated using the full covariance matrix. The cell e.s.d.'s are taken into account individually in the estimation of e.s.d.'s in distances, angles and torsion angles; correlations between e.s.d.'s in cell parameters are only used when they are defined by crystal symmetry. An approximate (isotropic) treatment of cell e.s.d.'s is used for estimating e.s.d.'s involving l.s. planes.
Refinement. Refinement of *F*^2^ against ALL reflections. The weighted *R*-factor *wR* and goodness of fit *S* are based on *F*^2^, conventional *R*-factors *R* are based on *F*, with *F* set to zero for negative *F*^2^. The threshold expression of *F*^2^ > σ(*F*^2^) is used only for calculating *R*-factors(gt) *etc*. and is not relevant to the choice of reflections for refinement. *R*-factors based on *F*^2^ are statistically about twice as large as those based on *F*, and *R*- factors based on ALL data will be even larger.

### Fractional atomic coordinates and isotropic or equivalent isotropic displacement parameters (Å^2^)

**Table:**

	*x*	*y*	*z*	*U*_iso_*/*U*_eq_	
C1	0.3065 (2)	0.63515 (13)	0.03856 (13)	0.0364 (3)	
H1A	0.3369	0.6320	−0.0485	0.044*	
H1B	0.4338	0.6149	0.0780	0.044*	
C2	0.1553 (2)	0.54630 (12)	0.11427 (12)	0.0324 (3)	
N3	0.01255 (17)	0.59887 (10)	0.18002 (10)	0.0314 (2)	
N4	0.05040 (17)	0.72620 (10)	0.16410 (10)	0.0304 (2)	
C5	−0.1328 (2)	0.81721 (13)	0.17228 (12)	0.0342 (3)	
H5	−0.0911	0.8976	0.1736	0.041*	
O6	−0.25382 (16)	0.84950 (11)	0.06661 (9)	0.0440 (3)	
C7	−0.1451 (2)	0.87381 (13)	−0.05137 (12)	0.0371 (3)	
C8	−0.2589 (3)	0.93655 (15)	−0.15442 (14)	0.0479 (4)	
H8	−0.3999	0.9630	−0.1409	0.057*	
C9	−0.1623 (3)	0.95933 (18)	−0.27641 (16)	0.0586 (5)	
H9	−0.2381	1.0010	−0.3458	0.070*	
C10	0.0466 (3)	0.92058 (17)	−0.29635 (15)	0.0560 (5)	
H10	0.1116	0.9354	−0.3792	0.067*	
C11	0.1589 (3)	0.86000 (14)	−0.19372 (13)	0.0449 (4)	
H11	0.3003	0.8352	−0.2079	0.054*	
C12	0.0646 (2)	0.83525 (12)	−0.06896 (12)	0.0347 (3)	
C13	0.1871 (2)	0.76641 (13)	0.04385 (12)	0.0330 (3)	
H13	0.2808	0.8222	0.0510	0.040*	
C14	0.1724 (2)	0.40903 (14)	0.11858 (15)	0.0418 (3)	
H14A	0.2925	0.3598	0.1589	0.063*	
H14B	0.1840	0.4044	0.0332	0.063*	
H14C	0.0517	0.3737	0.1665	0.063*	
C15	−0.2727 (2)	0.76809 (12)	0.29245 (12)	0.0329 (3)	
C16	−0.2086 (2)	0.75161 (17)	0.40981 (14)	0.0448 (4)	
H16	−0.0830	0.7752	0.4137	0.054*	
C17	−0.3307 (3)	0.70033 (17)	0.52093 (14)	0.0462 (4)	
H17	−0.2865	0.6890	0.5995	0.055*	
C18	−0.5184 (2)	0.66557 (13)	0.51636 (12)	0.0342 (3)	
C19	−0.5837 (2)	0.68431 (14)	0.39930 (13)	0.0374 (3)	
H19	−0.7103	0.6621	0.3952	0.045*	
C20	−0.4614 (2)	0.73595 (14)	0.28820 (13)	0.0381 (3)	
H20	−0.5070	0.7492	0.2096	0.046*	
C21	−0.6403 (2)	0.60087 (13)	0.63696 (13)	0.0361 (3)	
O7	−0.5895 (2)	0.58142 (13)	0.74163 (10)	0.0520 (3)	
O8	−0.80628 (16)	0.56450 (11)	0.61698 (10)	0.0454 (3)	
H8A	−0.8571	0.5179	0.6845	0.068*	

### Atomic displacement parameters (Å^2^)

**Table:**

	*U*^11^	*U*^22^	*U*^33^	*U*^12^	*U*^13^	*U*^23^
C1	0.0382 (7)	0.0364 (7)	0.0318 (6)	−0.0088 (5)	0.0038 (5)	−0.0084 (5)
C2	0.0362 (6)	0.0317 (6)	0.0286 (6)	−0.0074 (5)	−0.0017 (5)	−0.0077 (5)
N3	0.0364 (6)	0.0285 (5)	0.0281 (5)	−0.0088 (4)	−0.0002 (4)	−0.0065 (4)
N4	0.0362 (5)	0.0290 (5)	0.0256 (5)	−0.0104 (4)	0.0017 (4)	−0.0072 (4)
C5	0.0423 (7)	0.0310 (6)	0.0282 (6)	−0.0086 (5)	0.0016 (5)	−0.0082 (5)
O6	0.0434 (5)	0.0498 (6)	0.0273 (5)	−0.0018 (5)	−0.0005 (4)	−0.0009 (4)
C7	0.0514 (8)	0.0290 (6)	0.0273 (6)	−0.0098 (6)	−0.0004 (5)	−0.0034 (5)
C8	0.0585 (9)	0.0417 (8)	0.0359 (7)	−0.0042 (7)	−0.0097 (7)	−0.0015 (6)
C9	0.0890 (14)	0.0478 (9)	0.0316 (7)	−0.0059 (9)	−0.0143 (8)	−0.0009 (6)
C10	0.0856 (13)	0.0474 (9)	0.0279 (7)	−0.0115 (9)	0.0037 (8)	−0.0058 (6)
C11	0.0624 (9)	0.0362 (7)	0.0313 (7)	−0.0126 (7)	0.0087 (6)	−0.0072 (5)
C12	0.0507 (8)	0.0253 (6)	0.0270 (6)	−0.0126 (5)	0.0018 (5)	−0.0055 (4)
C13	0.0368 (6)	0.0331 (6)	0.0288 (6)	−0.0134 (5)	0.0036 (5)	−0.0079 (5)
C14	0.0464 (8)	0.0351 (7)	0.0449 (8)	−0.0074 (6)	−0.0004 (6)	−0.0149 (6)
C15	0.0403 (7)	0.0287 (6)	0.0276 (6)	−0.0059 (5)	0.0024 (5)	−0.0081 (5)
C16	0.0447 (8)	0.0600 (9)	0.0340 (7)	−0.0227 (7)	0.0018 (6)	−0.0147 (6)
C17	0.0524 (9)	0.0628 (10)	0.0270 (6)	−0.0203 (7)	−0.0002 (6)	−0.0139 (6)
C18	0.0396 (7)	0.0322 (6)	0.0289 (6)	−0.0055 (5)	0.0031 (5)	−0.0098 (5)
C19	0.0359 (7)	0.0410 (7)	0.0329 (6)	−0.0087 (5)	−0.0014 (5)	−0.0076 (5)
C20	0.0426 (7)	0.0417 (7)	0.0277 (6)	−0.0085 (6)	−0.0042 (5)	−0.0063 (5)
C21	0.0405 (7)	0.0337 (6)	0.0311 (6)	−0.0055 (5)	0.0038 (5)	−0.0097 (5)
O7	0.0637 (7)	0.0646 (7)	0.0285 (5)	−0.0204 (6)	0.0024 (5)	−0.0126 (5)
O8	0.0457 (6)	0.0506 (6)	0.0336 (5)	−0.0165 (5)	0.0032 (4)	−0.0029 (4)

### Geometric parameters (Å, °)

**Table:**

C1—C2	1.4934 (19)	C11—C12	1.3915 (19)
C1—C13	1.5249 (19)	C11—H11	0.930
C1—H1A	0.970	C12—C13	1.5101 (19)
C1—H1B	0.970	C13—H13	0.980
C2—N3	1.2730 (17)	C14—H14A	0.960
C2—C14	1.4776 (19)	C14—H14B	0.960
N3—N4	1.4178 (15)	C14—H14C	0.960
N4—C5	1.4335 (18)	C15—C20	1.376 (2)
N4—C13	1.4793 (17)	C15—C16	1.3839 (19)
C5—O6	1.4409 (17)	C16—C17	1.379 (2)
C5—C15	1.5047 (18)	C16—H16	0.930
C5—H5	0.980	C17—C18	1.384 (2)
O6—C7	1.3637 (17)	C17—H17	0.930
C7—C12	1.379 (2)	C18—C19	1.3783 (19)
C7—C8	1.387 (2)	C18—C21	1.4856 (19)
C8—C9	1.371 (2)	C19—C20	1.380 (2)
C8—H8	0.930	C19—H19	0.930
C9—C10	1.377 (3)	C20—H20	0.930
C9—H9	0.930	C21—O7	1.2053 (17)
C10—C11	1.375 (2)	C21—O8	1.3140 (18)
C10—H10	0.930	O8—H8A	0.820
			
C2—C1—C13	100.79 (11)	C7—C12—C13	120.90 (12)
C2—C1—H1A	111.6	C11—C12—C13	121.16 (14)
C13—C1—H1A	111.6	N4—C13—C12	111.46 (11)
C2—C1—H1B	111.6	N4—C13—C1	100.98 (10)
C13—C1—H1B	111.6	C12—C13—C1	112.93 (11)
H1A—C1—H1B	109.4	N4—C13—H13	110.4
N3—C2—C14	122.55 (13)	C12—C13—H13	110.4
N3—C2—C1	113.23 (12)	C1—C13—H13	110.4
C14—C2—C1	124.16 (12)	C2—C14—H14A	109.5
C2—N3—N4	108.71 (11)	C2—C14—H14B	109.5
N3—N4—C5	114.05 (10)	H14A—C14—H14B	109.5
N3—N4—C13	107.26 (9)	C2—C14—H14C	109.5
C5—N4—C13	114.20 (10)	H14A—C14—H14C	109.5
N4—C5—O6	113.73 (11)	H14B—C14—H14C	109.5
N4—C5—C15	112.11 (11)	C20—C15—C16	119.21 (13)
O6—C5—C15	106.99 (11)	C20—C15—C5	121.22 (12)
N4—C5—H5	107.9	C16—C15—C5	119.55 (13)
O6—C5—H5	107.9	C17—C16—C15	120.12 (14)
C15—C5—H5	107.9	C17—C16—H16	119.9
C7—O6—C5	115.49 (11)	C15—C16—H16	119.9
O6—C7—C12	122.64 (12)	C16—C17—C18	120.50 (13)
O6—C7—C8	116.04 (14)	C16—C17—H17	119.8
C12—C7—C8	121.29 (14)	C18—C17—H17	119.8
C9—C8—C7	119.60 (17)	C19—C18—C17	119.28 (13)
C9—C8—H8	120.2	C19—C18—C21	120.86 (13)
C7—C8—H8	120.2	C17—C18—C21	119.74 (13)
C8—C9—C10	120.15 (16)	C18—C19—C20	120.10 (13)
C8—C9—H9	119.9	C18—C19—H19	119.9
C10—C9—H9	119.9	C20—C19—H19	119.9
C11—C10—C9	119.92 (15)	C15—C20—C19	120.77 (13)
C11—C10—H10	120.0	C15—C20—H20	119.6
C9—C10—H10	120.0	C19—C20—H20	119.6
C10—C11—C12	121.10 (16)	O7—C21—O8	123.78 (13)
C10—C11—H11	119.4	O7—C21—C18	123.50 (14)
C12—C11—H11	119.4	O8—C21—C18	112.71 (12)
C7—C12—C11	117.93 (14)	C21—O8—H8A	109.5
			
C13—C1—C2—N3	−15.79 (15)	N3—N4—C13—C1	−29.67 (12)
C13—C1—C2—C14	166.98 (12)	C5—N4—C13—C1	−157.08 (11)
C14—C2—N3—N4	174.46 (11)	C7—C12—C13—N4	11.07 (17)
C1—C2—N3—N4	−2.82 (15)	C11—C12—C13—N4	−167.94 (12)
C2—N3—N4—C5	148.89 (11)	C7—C12—C13—C1	123.94 (13)
C2—N3—N4—C13	21.40 (13)	C11—C12—C13—C1	−55.08 (17)
N3—N4—C5—O6	−68.94 (13)	C2—C1—C13—N4	26.12 (12)
C13—N4—C5—O6	54.89 (15)	C2—C1—C13—C12	−93.01 (13)
N3—N4—C5—C15	52.60 (14)	N4—C5—C15—C20	−112.18 (15)
C13—N4—C5—C15	176.43 (10)	O6—C5—C15—C20	13.15 (17)
N4—C5—O6—C7	−44.40 (16)	N4—C5—C15—C16	66.12 (17)
C15—C5—O6—C7	−168.75 (11)	O6—C5—C15—C16	−168.55 (12)
C5—O6—C7—C12	17.97 (19)	C20—C15—C16—C17	1.7 (2)
C5—O6—C7—C8	−164.03 (12)	C5—C15—C16—C17	−176.64 (14)
O6—C7—C8—C9	−177.12 (14)	C15—C16—C17—C18	−0.4 (3)
C12—C7—C8—C9	0.9 (2)	C16—C17—C18—C19	−0.8 (2)
C7—C8—C9—C10	−0.3 (3)	C16—C17—C18—C21	175.18 (15)
C8—C9—C10—C11	−0.6 (3)	C17—C18—C19—C20	0.7 (2)
C9—C10—C11—C12	0.9 (3)	C21—C18—C19—C20	−175.28 (13)
O6—C7—C12—C11	177.24 (13)	C16—C15—C20—C19	−1.9 (2)
C8—C7—C12—C11	−0.7 (2)	C5—C15—C20—C19	176.45 (13)
O6—C7—C12—C13	−1.8 (2)	C18—C19—C20—C15	0.7 (2)
C8—C7—C12—C13	−179.70 (13)	C19—C18—C21—O7	179.54 (14)
C10—C11—C12—C7	−0.2 (2)	C17—C18—C21—O7	3.6 (2)
C10—C11—C12—C13	178.82 (14)	C19—C18—C21—O8	1.05 (19)
N3—N4—C13—C12	90.51 (12)	C17—C18—C21—O8	−174.89 (13)
C5—N4—C13—C12	−36.89 (15)		

### Hydrogen-bond geometry (Å, °)

**Table:**

*D*—H···*A*	*D*—H	H···*A*	*D*···*A*	*D*—H···*A*
O8—H8A···N3^i^	0.82	1.93	2.7356 (17)	168

Symmetry codes: (i) −*x*−1, −*y*+1, −*z*+1.

## References

[bb1] Palomer, A., Cabré, F., Pascual, J., Campos, J., Trujillo, M. A., Entrena, A., Gallo, M. A., Garcia, L., Mauleón, D. & Espinosa, A. (2002). *J. Med. Chem.* **45**, 1402–1411. 10.1021/jm010458r 11906281

[bb2] Sheldrick, G. M. (2008). *Acta Cryst.* A**64**, 112–122. 10.1107/S0108767307043930 18156677

[bb3] Siemens (1991). *XSCANS* Siemens Analytical X-ray Instruments Inc., Madison, Wisconsin, USA.

[bb4] Spek, A. L. (2003). *J. Appl. Cryst.* **36**, 7–13.

[bb5] Subbaramaiah, K., Norton, L., Gerald, W. & Dannenberg, A. J. (2002). *J. Biol. Chem.* **277**, 18649–18659. 10.1074/jbc.M111415200 11901151

[bb6] Světlík, J., Pronayova, N. & Kubista, J. (2005). *J. Heterocycl. Chem.* **42**, 1143–1147.

